# Scaling mimesis: Morphometric and ecomorphological similarities in three sympatric plant-mimetic fish of the family Carangidae (Teleostei)

**DOI:** 10.1371/journal.pone.0194437

**Published:** 2018-03-20

**Authors:** Alexya Cunha de Queiroz, Marcelo Vallinoto, Yoichi Sakai, Tommaso Giarrizzo, Breno Barros

**Affiliations:** 1 Laboratory of Evolution, Federal University of Pará, Bragança campus, Bragança, Brazil; 2 CIBIO-InBIO, Centro de Investigação em Biodiversidade e Recursos Genéticos, Campus Agrário de Vairão, Universidade do Porto, Vairão, Portugal; 3 Laboratory of the Biology of Aquatic Resources, Graduate School of Biosphere Science, Hiroshima University, Higashi-Hiroshima, Japan; 4 Universidade Federal do Pará, Instituto de Ciências Biológicas, Laboratório de Biologia Pesqueira e Manejo dos Recursos Aquáticos, Grupo de Ecologia Aquática – GEA, Belém, PA, Brazil; 5 Integrated Laboratory of Animal Behavior, Federal Rural University of Amazonia, campus of Capanema, Capanema, Brazil; Universidade Federal do Rio de Janeiro, BRAZIL

## Abstract

The mimetic juveniles of a number of carangid fish species resemble plant parts floating near the water surface, such as leaves, seeds and other plant debris. The present study is the first to verify the morphological similarities and ecomorphological relationships between three carangids (*Oligoplites saurus*, *Oligoplites palometa* and *Trachinotus falcatus*) and their associated plant models. Behavioral observations were conducted in the estuary of Curuçá River, in northeastern Pará (Brazil) between August 2015 and July 2016. Individual fishes and associated floating objects (models) were sampled for comparative analysis using both geometric and morphometric approaches. While the mimetic fish and their models retain their own distinct, intrinsic morphological features, a high degree of morphological similarity was found between each fish species and its model. The morphometric analyses revealed a general tendency of isometric development in all three fish species, probably related to their pelagic habitats, during all ontogenetic stages.

## Introduction

Coastal ecosystems, especially estuaries, are regarded as excellent nursery environments for many marine organisms, in particular fish [1–3]. In the tropical zone, mangrove ecosystems constitute an especially important, and highly productive environment [4] used as spawning grounds and nurseries (feeding and shelter) by an enormous variety of marine organisms [5–7]. Estuarine environments are regarded attractive to juvenile fish, presenting lower predation risks and a much higher availability of food and feeding sites [3, 5, 8, 9].

Behavioral strategies associated with mimetism and camouflage in fish have been widely studied [10–15], and the phenomenon is relatively well documented in both freshwater and marine species [16–20]. However, the specific mechanisms involved in this phenomenon are still poorly understood, especially in the case of plant mimesis, due to the difficulties of conducting systematic observations under field conditions [19, 21]. The analysis of the anti-predatory behavior and coloration in fish can provide useful insights for the understanding of the ecological and evolutionary relationships among different species, e.g., fish-plant mimics [10, 22–25], given that these behavior patterns have been associated with the population dynamics of a number of different fish species [26].

Breder [10, 22] first discussed the evolutionary importance of this type of behavior, based on observations of a number of different fish species, including mangrove-dwelling forms, such as the carangids *Oligoplites saurus* and *Trachinotus falcatus*, which were observed drifting in the water, together with debris (leaves, seeds) derived from the mangrove forest.

The family Carangidae is a cosmopolitan group that inhabits tropical and warm-temperate waters, and contributes approximately 5% of commercial fishery catches worldwide [27, 28]. The natural history of the carangids is still poorly understood, even in the case of the commercially important species, and particularly for the juvenile stages [28, 29]. While some species are known to be plant-mimetic while in the juvenile stage, only a few descriptive data are available [10, 22, 30, 31].

The analysis of the shape of an organism can provide important insights into its ecological and evolutionary characteristics [32–34]. Recent studies have employed complex tools, such as geometrical morphometric modeling and ecomorphological analyses, to understand the developmental and ontogenetic dynamics of the body shape of a number of organisms, including mimetic fish [15, 21, 35, 36].

In the present study, these tools were employed to estimate the developmental stage at which the juveniles of the sympatric carangids *Oligoplites palometa*, *O*. *saurus* and *Trachinotus falcatus* cease their mimetic behavior, by testing the following null hypotheses: (1) mimetic juveniles and their associated plant models form distinct clusters, sharing no morphometric features, and (2) juvenile fish (mimetics) and late-juveniles/adults (non-mimetics) present similar morphometric patterns during growth. A brief description of the mimetic behavior of the study species is presented, followed by the analysis of morphometric patterns related to the plant mimesis.

## Material and methods

### Study area and sampling procedures

**Fig 1:**
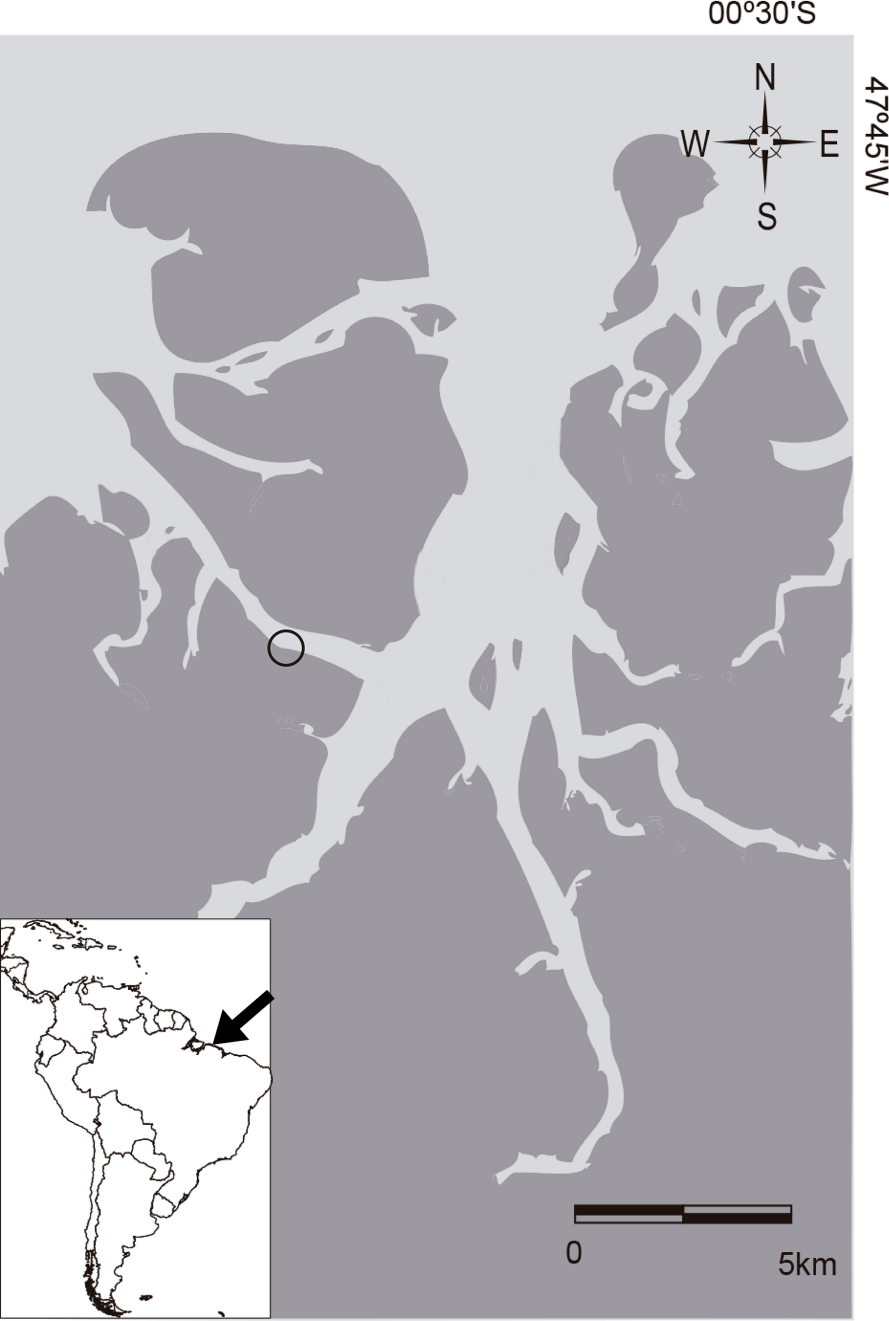
Map of the sampling site at Curuçá, PA. Map of the sampling site at Curuçá, Northeastern PA.

Data were collected monthly between June 2015 and June 2016 in the mangrove estuary of the Curuçá River, in northeastern Pará, Brazil (0°10’S, 47°50’W; Fig 1). This estuary covers an area of 200 km^2^, including the village of Abade, a well-preserved environment that has been designated as an extractive reserve, a sustainable-use conservation unit, by the Brazilian Ministry of the Environment [37]. The study consists in a macro-tidal, predominantly marine system, with very low freshwater influx, covering approximately 116Km^2^ of mangrove forests, comprised by three main species: *Rhizophora mangle*, *Laguncularia racemosa* and *Avicennia germinans*. Fluctuant leaves, seeds, fruis, flowers and propagules of mangrove plants are highly abundant in the water surface during the round year. Salinity levels are homogeneous in the entire area, presenting no spatial differences among pH, turbidity, or oxygen levels. During the rainy season, turbidity levels are higher, with lower levels during the peak of dry season (October-December). Annual mean precipitation is 2526mm^3^) [38, 39].

Juvenile mimetic carangids were observed in association with floating plant debris (leaves, seeds, flowers and petioles). Juvenile mimetic fishes (hereafter, mimetics) and associated plant parts (hereafter, models) were collected using 20 cm x 20 cm hand nets. The fish were euthanized using a stock solution containing 5 ml of 95% eugenol in 1 L of ethanol, diluted (20 ml per liter) in the water containing the specimens, which were then preserved in 70% ethanol. The models were sorted in the field and classified preliminarily as leaves, seeds, petioles and flowers, and then dried for final identification and measurement in the laboratory. None of the surveyed species is considered threatened, and all sampling activities were performed under permission of the ICMBio—Brazilian Institute for Conservation of Biodiversity (license SISBIO #54888–1), which include animal welfare and ethics according to international standards.

**Fig 2:**
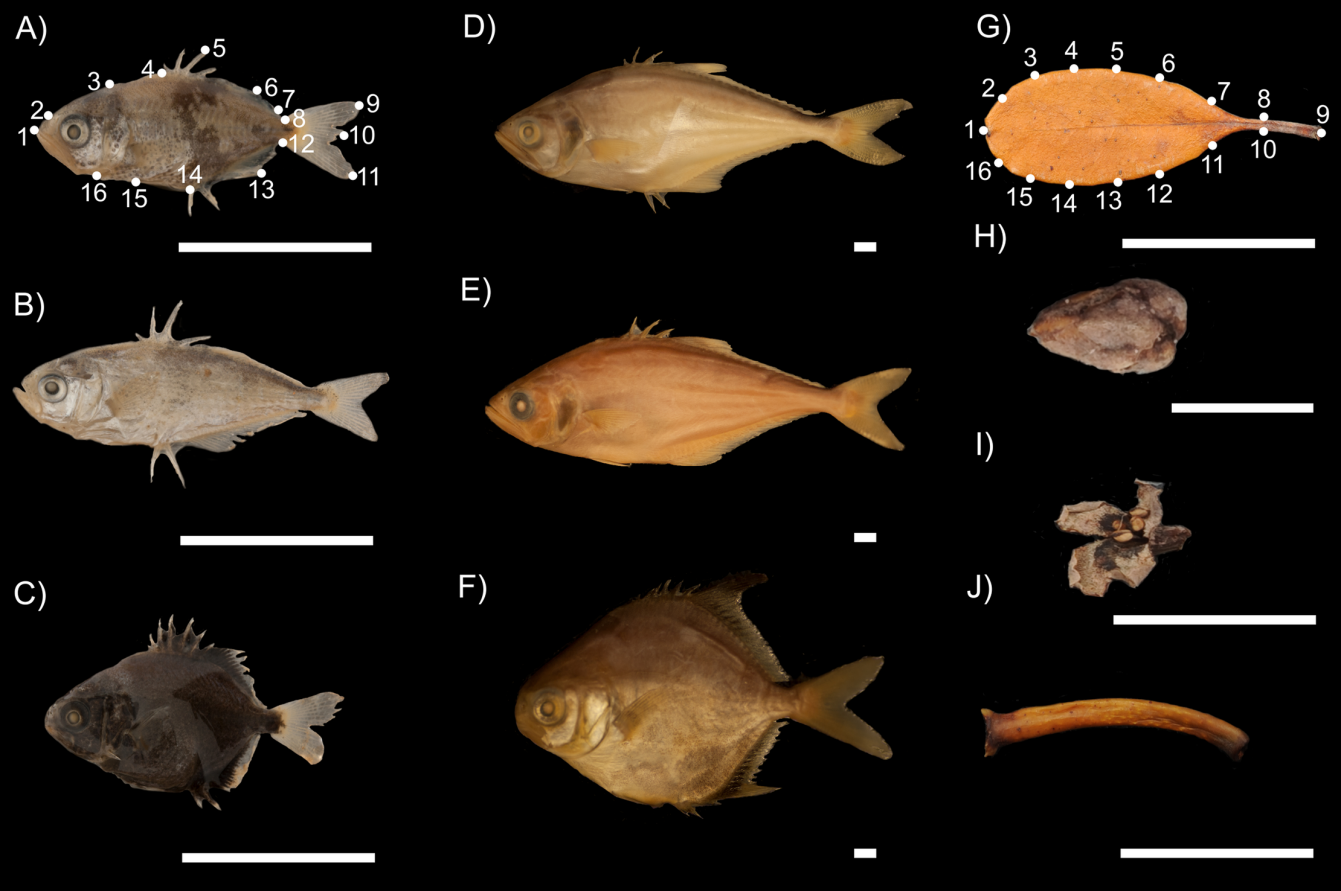
Mimetic carangid fish species associated to their respective plant models. Mimetic carangid fish species associated to their respective plant models. Three species of mimetic Carangidae associated to their respective plant models, where (A) and (D) denote respectively juvenile and adult *Oligoplites palometa*; (B) and (E) juvenile and adult *O*. *saurus*; (C) and (F) juvenile and adult *Trachinotus falcatus*; (G) *Rhizophora mangle* leaf; (H) *Laguncularia racemosa* seed; (I) *L*. *racemosa* flower; and (J) unspecified mangrove petiole.

All data were collected during neap tides, following a complete tidal cycle (flood-high-ebb-low tides) in order to standardize the amount of biomass collected for both mimetics and models. An attempt was made to sample similar numbers of mimetics and models. We collected 136 *Oligoplites palometa* (Mean ± SD Total Length [TL] = 1.00±0.25 cm, Fig 2A), 8 *Oligoplites saurus* (TL = 2.12±0.41 cm Fig 2B), and 9 *Trachinotus falcatus* (TL = 1.52±0.11 cm, Fig 2C). To complement this sample, we included specimens (mostly late juveniles and adults) kindly provided by the Goeldi Museum (MPEG), in Belém, Pará, Brazil. These specimens included 20 *O*. *palometa (*TL = 9.18±6.85 cm; Fig 2D), 34 *O*. *saurus* (TL = 8.63±4.35 cm; Fig 2E), and 4 *T*. *falcatus* (TL = 5.66±0.17 cm; Fig 2F). These samples were collected in the estuary of the Caeté River, on the Bragança Peninsula (Pará, Brazil 01°03' S, 46°45' W), located in the vicinity of our study area, as for the most r-strategist fish species belong to the same general population [40, 41]. Only well-preserved specimens, with intact peripheral structures, were used in the analyses, in order to avoid the inclusion of any deformations that would bias the findings, as indicated by Valentin et al. [42].

A total of 169 models were collected, including 31 leaves (TL = 3.38±2.19 cm; Fig 2G), 60 seeds (TL = 1.04±0.69 cm; Fig 2H), 49 flowers (TL = 0.44±0.63 cm; Fig 2I), and 89 petioles (TL = 3.57 ± 1.42 cm, Fig 2J).

### Geometric morphometrics and ecomorphology

High resolution digital photographs were taken of the left lateral view of the mimetics and models on a black background, using a Nikon D700 camera equipped with an AF-S 60mm immersive lens and a stand table with a reference scale of 1 cm. The left lateral view of the models was defined as the dorsal view of the leaf with the petiole oriented to the right. Artificial light was used to avoid shading the morphological structures.

Sixteen landmarks (LMs) were established for the analyses of both the mimetics and their respective models, using ImageJ v. 1.47 [43], with a protocol adapted from [15]. Homologous LMs were used for the different mimetics, based on morphological structures related to the mimetic behavior (i.e., unpaired fins), covering the whole lateral body profile (left oriented), including peripheral structures, such as the tips of the rays and spines of all the fins (Fig 2A, Table 1).

**Table 1:** List of landmarks used for the geometric morphometric analysis.

Landmark	Description of landmark
1	Tip of the snout
2	Nasal cavity
3	Posterior limit of supra-occipital
4	Anterior insertion of dorsal fin
5	Edge of last hard spine
6	Insertion of soft rays
7	Maximum height of dorsal fin
8	Posterior insertion of dorsal fin
9	Upper limit of caudal fin
10	Hypural joint
11	Lower limit of caudal fin
12	Posterior insertion of anal fin
13	Maximum height of anal fin
14	Anterior insertion of anal fin
15	Insertion of pelvic fin
16	Lower occipital edge

Anatomic landmarks in fish were chosen in order to cover the peripheral shape area.

For the models, 16 equidistant semi-landmarks (sLMs) were established per individual, employing the grid tool available in ImageJ, also covering the whole lateral profile of each plant part analyzed (Fig 2G). The analysis of the correlation between the LMs in the mimetics and the corresponding sLMs in the models was based on the assumption that the probability of sharing a similar distribution of landmarks was determined by chance, so that the morphometric comparisons between the fish and the models were not intended to analyze homologous patterns, because we were interested in the similarities in shape shared randomly between the mimetic fish and their respective models, found in the same environment [15]. The raw coordinates of both LMs and sLMs were then fed into MorphoJ v. 1.02n [44], for preliminary adjustments, including the Procrustes and the creation of the data matrix.

Standard ecomorphological indices were also calculated for both mimetics and models, in order to establish predictions on swimming patterns and the position of the organisms in the water column [45–47], as well as to formulate assumptions on the position and drifting patterns of the models. Analogical callipers (0.1 mm precision) were used to determine four ecomorphological indices:

1. Compression Index (CI), which reflects the position of the fish in the water column. This index is calculated by dividing the maximum body depth by the maximum body width. High values for this index indicate a laterally compressed organism, typically adapted to pelagic environments [47];
2. Relative Body Depth (RBD), which reflects the capacity of the fish for vertical maneuvers. The index is calculated by dividing the maximum body depth by its body length. Low RBD values indicate a relatively elongated organism, and higher values are indicative of greater maneuverability [46];
3. Caudal Peduncle Compression Index (CPC), which indicates swimming rates. The index is calculated by dividing the depth of the peduncle at its midpoint by its width at the same point. Swimming rates are inversely related to the value of the CPC [46];
4. Index of Ventral Flattening (IVF), which reflects ecosystem hydrodynamics. The index is calculated by dividing the maximum midline depth by the maximum body depth. Low IVF values usually indicate organisms inhabiting high hydrodynamic habitats.

These four indices were selected for the present study because they could also be measured in the models. Adjustment was only necessary for the CPC, which was calculated in the models based on the measurements of the petioles, rather than the caudal peduncle.

### Growth patterns in the carangids

The multivariate analysis of allometric signals was based on the fish LMs (mimetics and non-mimetics), run in Geomorph V. 3.0.3 [48]. This analysis determines whether the study species grow allometrically or isometrically. The results were then compared with the growth patterns observed in the data on the ecomorphological indices, based on the univariate analyses described below.

### Statistical analyses

The log-centroid landmark values were evaluated for normality using the Shapiro-Wilk with the *post hoc* application of Student’s *t* (W = 0.93, *t* = 3.18, *P* < 0.001). The ecomorphological indices were also normally distributed (CI: W = 0.81 for mimetics and W = 0.44 for models, *t* = -3.46; RBD: W = 0.77 for mimetics and W = 0.69 for models, *t* = -6.24; CPC: W = 0.99 for mimetics and W = 0.48 for models, *t* = 8.49; IVF: W = 0.94 for mimetics and W = 0.96 for models, *t* = 9.46; *P* values were < 0.001 in all cases).

The geometric morphometric analyses were run in Geomorph V. 3.0.3 [48], through a Procrustes Generalized Analysis (GPA) for the visualization of the distribution of the LMs and sLMs, followed by a Principal Components Analysis (PCA) with an Analysis of Variance (ANOVA), using the pooled data for both LMs and sLMs, in order to assess the correlations between the mimetics and the models.

A Principal Coordinates Analysis (PCO), based on Bray-Curtis similarity, was applied to obtain a graphical unconstrained ordination of the mimetic and model samples, using the square-root transformed ecomorphological indices. The PCO, otherwise known as metric multidimensional scaling, is an ordination procedure that provides a direct projection of the distribution of the points (samples) in space, as defined by their dissimilarities [49].

To assess the similarity between the fish and plant (model) groups based on the ecomorphological indices, a cluster analysis was run using the standard agglomerative method, based on the distances between the centroids of the mimetic and model samples. The resulting dendrogram was linked to a shade plot to display the contribution of each ecomorphological index to each treatment.

Growth patterns were assessed by geometric morphometrics, through the allometry function in Geomorph V. 3.0.3 [48], using the fish LM data, and by single linear regression analyses, using the ecomorphological indices. The single linear regression analyses were followed by ANOVAs, which used each index as a dependent variable in relation to fish length (TL: cm). The F values are presented with two degrees of freedom (DF) in all cases, with adjusted R^2^ values, followed by the respective probability (*P*).

The GPAs, PCAs, single linear regressions and ANOVAs were run in the specific packages of the R environment, v. 3.3.3 [50]. The PCOs, cluster analyses, and shade plots were obtained using PRIMER 7 [51]. All raw data used in the statistical analyses are provided (S1 File).

## Results

### Mimetic behavior in the three carangid fish species

Similar plant-mimetic behavioral patterns were observed in all three study species, in close association with their models. *Oligoplites saurus* and *O*. *palometa* were often observed drifting alongside their models, adopting both a c-fold conformation, with the head pointing toward the bottom, and an s-shape conformation, with the whole body parallel to the water surface. *Trachinotus falcatus*, by contrast, presented only the c-fold body conformation, usually during twisting movements while maneuvering close to the water surface. Most of the time, this species is observed swimming parallel to the water surface, usually following drifting plant debris, according to the flow of the water.

### Morphometrics

**Fig 3:**
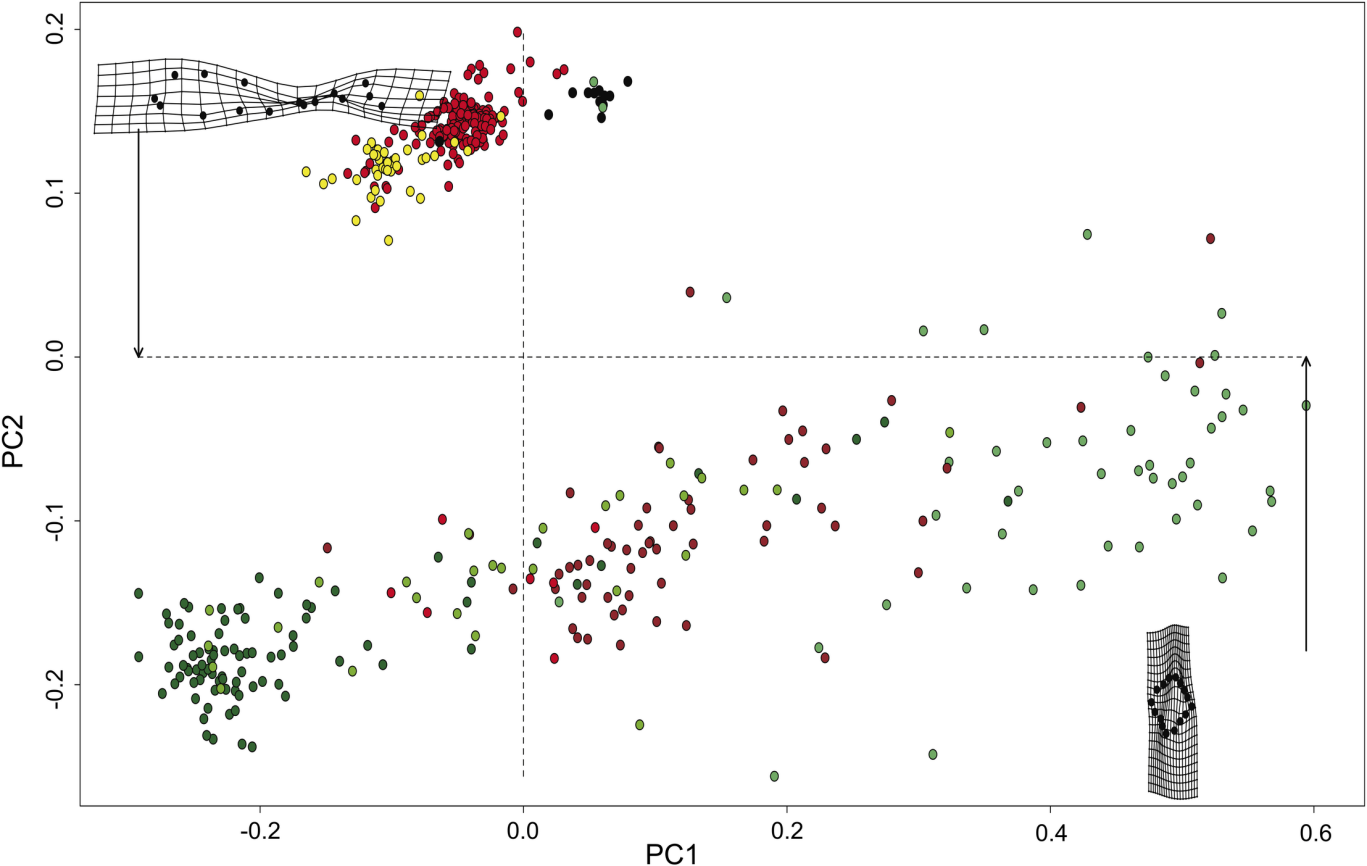
Morphospace showing morphometric similarities shared by mimetic fish and plant models. PCA analysis showing the morphometric similarities shared among the three species observed to their respective models, where yellow plots denote *O*. *saurus*; red plots denote *O*. *palometa* and black plots denote *T*. *falcatus*. Models are represented by different shades of green for leaves, seeds and flowers, with petioles are denoted in brown.

The results of the PCAs indicate a degree of correlation between the morphometric data of the mimetics and models (F = 166.73, DF = 6, 431, *P* < 0.001), with a difference of less than 20% being observed between fish and plant groups, as explained by the first axis, PC1 (Fig 3). Despite the tendency for mimetics and models to cluster in different groups, there is still a degree of mixture of the mimetics (*O*. *saurus* in yellow, *O*. *palometa* in red, *T*. *falcatus* in black) and models (different shades of green for leaves, seeds and flowers, and petioles in brown) in both clusters (Fig 3).

**Fig 4:**
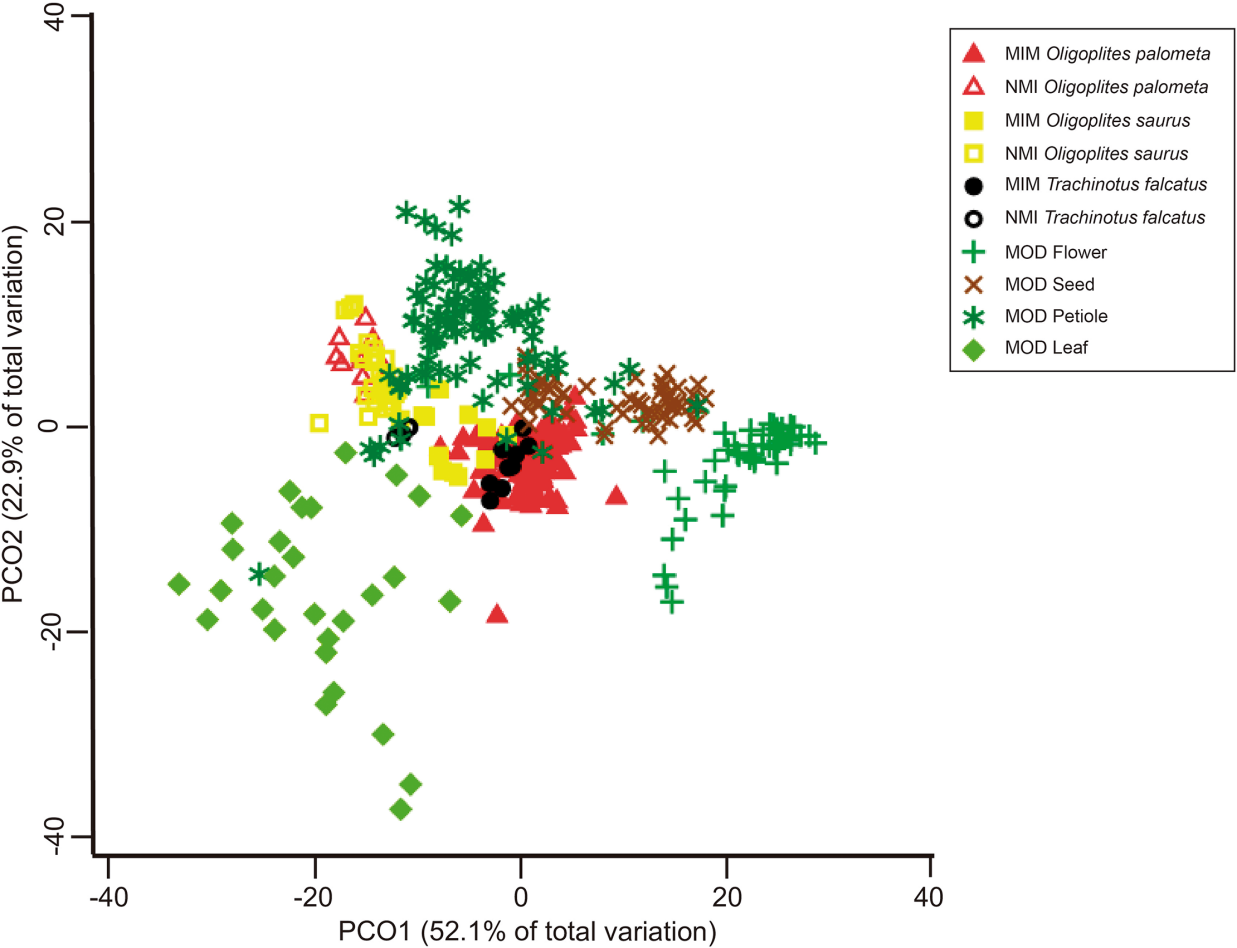
PCO analysis showing dissimilarity levels observed for fish and models. Principal coordinates analysis showed how different are mimetic and adult fish, according to the analyzed ecomorphological indexes, and how close (similar) are mimetic fish with their respective associated models, where filled symbols denote non-mimetic adult fish, and blank symbols denote mimetic juvenile fish. *O*. *palometa* are represented by yellow symbols; *O*. *saurus* by red symbols and *T*. *falcatus* by black symbols. Models are represented by different shades of green for leaves, seeds and flowers, with petioles are denoted in brown.

The Principal Coordinates Analysis (PCO) indicated a clear separation between the mimetics and models (Fig 4). Overall, 75% of the variation in the data was explained by the first two axes. The juvenile (mimetic), and adult (non-mimetic) fish were delineated in two distinct groups along the first PCO axis. Leaf and flower samples were arranged to the right and left of the first PCO axis, whereas the petioles and seeds were located towards the top central of the plot close of the mimetic samples.

**Fig 5:**
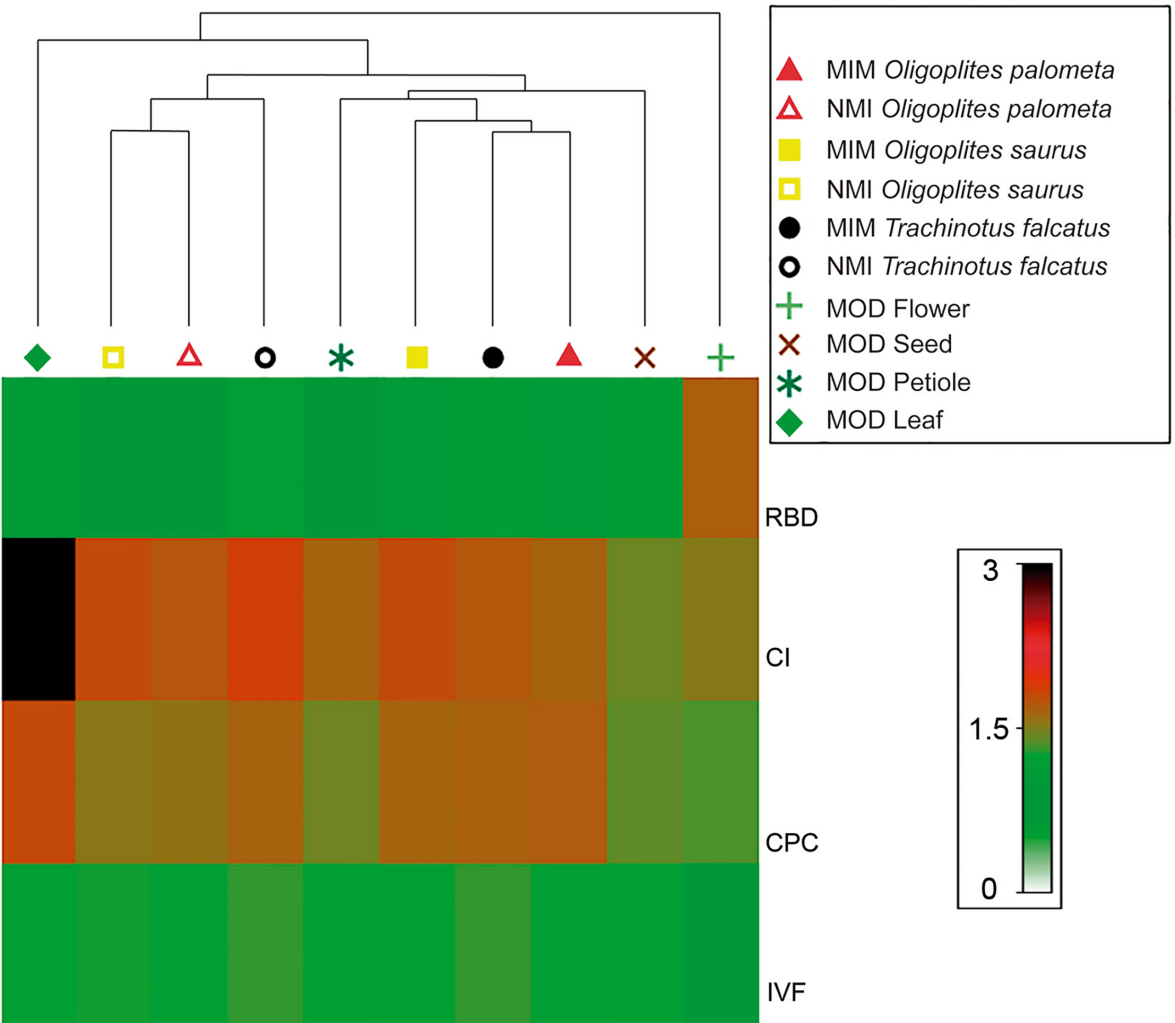
Cluster analysis and shade plot showing similarity degrees of mimetic fish and associated models. Cluster associations separating clearly mimetic and non-mimetic fish, and showing the similarity levels observed among mimetic fish and their respective associated models, where filled symbols denote non-mimetic adult fish (NMI), and blank symbols denote mimetic juvenile fish (MIM). *O*. *palometa* are represented by yellow symbols; *O*. *saurus* by red symbols and *T*. *falcatus* by black symbols. Models (MOD) are represented by different shades of green for leaves, seeds and flowers, with petioles are denoted in brown. In the shade plot, RBD refers to “Relative Body Depth”; CI, “Compression Index”; CPC, “Caudal Peduncle Compression Index”; and IVF, “Index of Ventral Flattening”, respectively.

The cluster analysis and shade plot derived from the centroids of the ecomorphological indices of the mimetics and models revealed that the mimetic juvenile stages of the three fish species were more similar to the petiole and seed models than the adult fish stages (Fig 5). Leaves and flowers, with the highest CI and RBD values, respectively, were the most dissimilar from both the other models and the mimetics.

### Growth patterns

**Fig 6:**
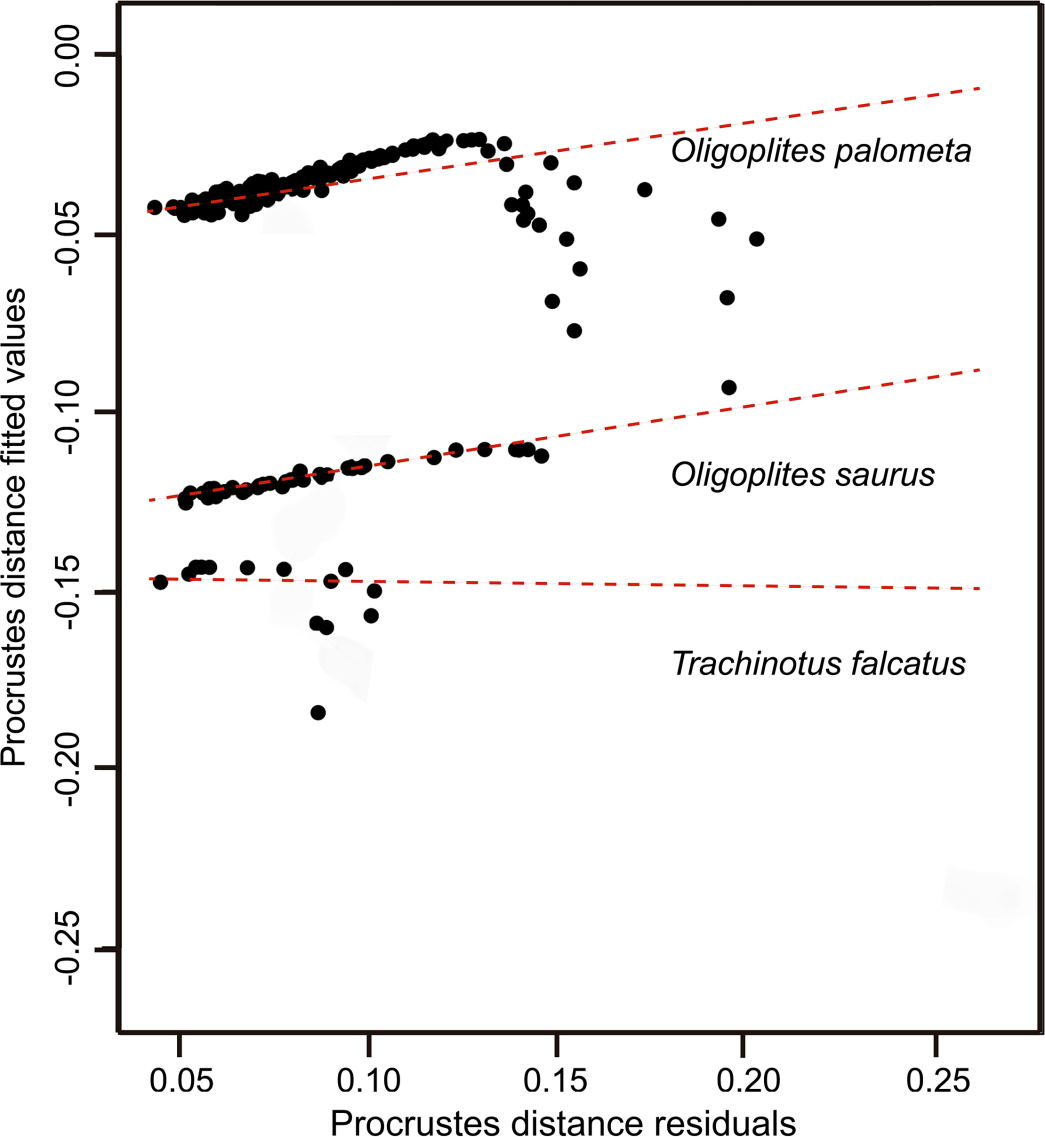
Isometric growth patterns observed for three carangid species according to LM data. A general isometric pattern was observed for all three Carangid species during mimetic juvenile stages.

**Fig 7:**
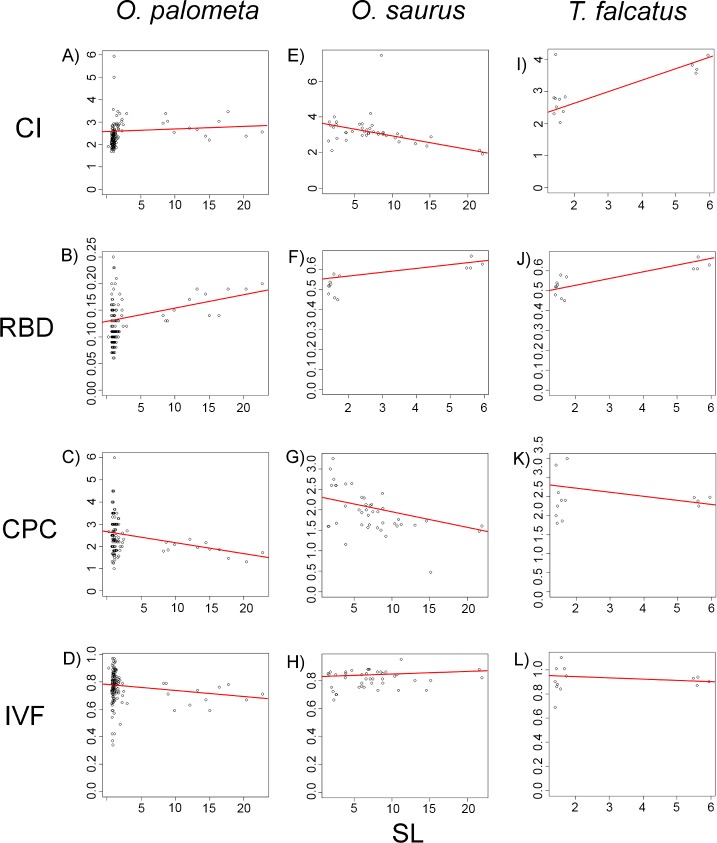
Growth patterns observed according to the ecomorphological indexes. All ecomorphological indexes employed showed similar isometric growth patterns for all three mimetic carangid species. Fish species are divided in columns, where (A-D) show growth analyses for *O*. *palometa*, (E-H) for *O*. *saurus*, and (I-L) for *T*. *falcatus*, where RBD refers to “Relative Body Depth”; CI, “Compression Index”; CPC, “Caudal Peduncle Compression Index”; IVF, “Index of Ventral Flattening”; and SL (cm) refers to “Standard Length”, respectively.

The morphology of the three study species varied little during growth, and followed a general isometric growth pattern, as shown by both the allometric analysis of the LMs and the single linear regressions based on the variance of the ecomorphological indices recorded through the course of ontogeny. The standard growth curves based on the LM data were highly similar in the three study species (F = 5.39, DF = 1, 271; R^2^ = 0.023; *P* < 0.01) (Fig 6). The regression analyses of the ecomorphological indices also indicated similar isometric patterns in the three species (Fig 7A–7L).

All three species presented high CI values (*O*. *palometa* CI *=* 2.32±0.51, *O*. *saurus*, CI *=* 3.19±0.81; *T*. *falcatus*, CI *=* 3.06±0.69) throughout their ontogeny, and all three species grew isometrically (*O*. *palometa*, F = 17.51, DF = 1, 213, R^2^ = 0.07, *P* < 0.001; *O*. *saurus*, F = 4.13, DF = 1, 40, R^2^ = 0.07, *P* < 0.001; *T*. *falcatus*, F = 11.05, DF = 1, 11 DF, R^2^ = 0.46, *P* < 0.01, Fig 7A–7C).

The RBD values recorded for all three study species were relatively low in all three species (*O*. *palometa*, RBD = 0.35±0.05, *O*. *saurus*, RBD = 0.30±0.02; *T*. *falcatus*, RBD = 0.55±0.06), indicating an overall tendency for an elongated shape, which is also characteristic of isometric development (*O*. *palometa*, F = 17.51, DF = 1, 213, R^2^ = 0.04, *P* < 0.001; *O*. *saurus*, F = 17.42, DF = 1, 40, R^2^ = 0.28, *P* < 0.001; *T*. *falcatus*, F = 20.65, DF = 1, 11, R^2^ = 0.62, *P* < 0.001, Fig 7D–7F).

All three species presented high CPC values (*O*. *palometa*, CPC = 2.59±0.83, *O*. *saurus*, CPC = 1.94±0.51; *T*. *falcatus*, CPC = 2.44±0.47), that is, a highly compressed caudal peduncle, which is preserved throughout development (*O*. *palometa*, F = 11.6, DF = 1, 213, R^2^ = 0.04, *P* < 0.001; *O*. *saurus*, F = 16.69, DF = 1, 40, R^2^ = 0.27, *P* < 0.001; *T*. *falcatus*, DF = 0.023, DF = 1, 11, R^2^ = -0.08, *P* > 0.05, Fig 7G–7I).

The IVF values recorded in the present study were low in all three species (*O*. *palometa*, IVF = 0.76±0.10, *O*. *saurus*, IVF = 0.81±0.06; *T*. *falcatus*, IVF = 0.91±0.09), indicating these fish are adapted to survival in highly hydrodynamic waters throughout their life cycle (*O*. *palometa*, F = 4.64, DF = 1, 213, R^2^ = 0.01, *P* < 0.05; *O*. *saurus*, F = 1.58, DF = 1, 40, R^2^ = 0.01, *P* > 0.05; *T*. *falcatus*, F = 0.0003145, DF = 1, 11, R^2^ = -0.09, *P* > 0.05, Fig 7J–7L).

## Discussion

The results of the present study have shown that the mimetic fish species share many morphological similarities with their respective plant models, as observed previously in juvenile *O*. *palometa* and *T*. *falcatus*, which have been described as mimetics of the seeds of the white mangrove, *Laguncularia racemosa* [10, 30]. Similar patterns of mimesis have been described in a number of freshwater fish species [20, 52, 53], although the similarities between the fish and their plant models were not analyzed statistically. The findings of the present study indicate that the body shape of the fish has a strong influence on the ecology of the organism, with the juveniles of all three species adopting mimetic behavioral strategies to take advantage of their morphological similarities with their plant models to avoid the attention of predators, as similarly observed in other mimetic species [19].

Plant mimesis in fish involves not only similarities in shape between the fish and its model, but also in coloration and behavior, as observed originally by Breder [10, 22], Uchida [23], and more recently by several other authors [14, 17, 19, 37, 54]. However, none of these previous studies has focused on the specific similarities between the general morphology of the mimetic fish and their plant models. The study species analyzed here are not only highly similar to their plant models in morphological terms, but also present similar drifting behavior patterns, as observed in round batfish *Platax orbicularis* [19]. This degree of association between mimetics and models indicates a dependence on the availability of plant debris at the surface of the water [15]. The morphological, morphometric and behavioral similarities shared by the mimetics and their models may thus be vital for survival in this micro-habitat, at least during the early life stages, allowing the fish to both avoid predators and forage more efficiently [14, 19, 21, 31, 37].

A general tendency of isometric growth was also found in all three species, contrasting with the patterns observed in the species *Chaetodipterus faber*, *P*. *orbicularis* and *Diplodus vulgaris* [21, 36]. These authors concluded that allometric growth may be essential for the pre-settlement of fish species that occupy multiple habitats during their different growth stages. However, the carangid species analyzed in the present study have pelagic habits throughout their entire life cycle, and are invariably found near the water surface [55], which suggests a functional link with their isometric development.

All the ecomorphological indices used in the present study were selected due to their insightful value for the interpretation of ecological processes, based on Gatz [46], Gibran [47], Hora [56] and Watson & Balon [57]. The high CI values observed in all three species indicate a high degree of lateral compression, which is linked to the occupation of habitats near the water surface [57]. The low RBD values observed, however, indicate that all three species are relatively elongated. Gatz [46] proposed that low RBD values combined with high CPC indices are correlated negatively with the ability to maneuver vertically, although this did not appear to be the case here, at least in the *Oligoplites* species, which were observed assuming a c-bound shape while drifting, and then suddenly switching to their natural elongated conformation [10]. Barros et al. [37] observed a similar behavioral pattern associated with zooplanktivory in the plant-mimetic juveniles of *Platax orbicularis*.

All three species also had high CPC values, which indicate a compressed caudal peduncle, which may be characteristic of less active swimmers (see [46]). While determining the swimming capability of the three species was not an objective of the present study, the field observations indicated that all three are capable swimmers, corroborating the observations of Leis [58], who affirms that the larval and juvenile fish are extremely active, and are capable of swimming considerable distances.

The assumption that juvenile aquatic organisms, especially fish, are mostly transported passively [59–61] may thus not be entirely correct. Considering the combination of biotic (e.g., the availability of floating plant debris) and abiotic (e.g., the tidal regime) factors usually associated with the dispersal of aquatic organisms [62], the results of the present study indicate that all three species are highly versatile in their swimming capacity, and their behavioral and ecological adaptability.

The low IVF values recorded here, in turn, indicate that these organisms are able to remain motionless even in highly hydrodynamic environments (i.e., subject to strong waves and winds), as observed by Hora [56]. While more field studies will be necessary for a more detailed understanding of the dispersal mechanisms of the three carangid species, the observed combination of ecomorphological traits that contribute to both their mimetic behavior and swimming capacity may provide important insights.

Strictly morphological studies are rarely able to cover the whole natural history of an organism [34]. The present study has provided important insights into the spatial distribution of the study species during their development and life cycle, in particular in relation to their plant-mimetic behavior, and the intimate association with the microhabitat of the water surface, where the plant models are also found. While the statistical tools employed in the present study highlighted the similarities between the mimetic species and their models, they fall short of elucidating the mathematical similarities between the fish and their associated floating plant parts. Future studies, combining a number of complementary approaches, including behavioral analysis, a more refined analysis of morphological and coloration patterns may provide more systematic insights into the dependence of the mimetic fish on their associated models.

## Supporting information

S1 File Raw data used in the present study, including landmarks and ecological indices. (XLSX)

## References

[1] Blaber SJM. Fishes and fisheries in tropical estuaries: the last 10 years. Estuarine, Coastal and Shelf Science. 2013;135:57–65.

[2] Nagelkerken I, Sheaves M, Baker R, Connolly RM. The seascape nursery: a novel spatial approach to identify and manage nurseries for coastal marine fauna. Fish and Fisheries. 2015;16(2):362–71.

[3] Blaber SJM, Barletta M. A review of estuarine fish research in South America: what has been achieved and what is the future for sustainability and conservation? Journal of Fish Biology. 2016;89(1):537–68. doi: 10.1111/jfb.12875 26864605 10.1111/jfb.12875

[4] Hogarth PJ. The biology of mangroves and seagrasses: Oxford University Press; 2015.

[5] Laegdsgaard P, Johnson C. Why do juvenile fish utilise mangrove habitats? Journal of Experimental Marine Biology and Ecology. 2001;257(2):229–53. 11245878 10.1016/s0022-0981(00)00331-2

[6] Mumby PJ, Edwards AJ, Arias-González JE, Lindeman KC, Blackwell PG, Gall A, et al Mangroves enhance the biomass of coral reef fish communities in the Caribbean. Nature. 2004;427(6974):533–6. doi: 10.1038/nature02286 14765193 10.1038/nature02286

[7] Elliott M, Hemingway KL. Fishes in estuaries: John Wiley & Sons; 2008.

[8] Blaber SJM, Blaber TG. Factors affecting the distribution of juvenile estuarine and inshore fish. Journal of Fish Biology. 1980;17(2):143–62.

[9] Robertson AI, Duke NC. Recruitment, growth and residence time of fishes in a tropical Australian mangrove system. Estuarine, Coastal and Shelf Science. 1990;31(5):723–43.

[10] Breder CM. An analysis of the deceptive resemblances of fishes to plant parts: with critical remarks on protective coloration, mimicry and adaptation. Bulletin of the Bingham Oceanographic Collection. 1946;10:1–49.

[11] Skelhorn J, Rowland HM, Ruxton GD. The evolution and ecology of masquerade. Biological Journal of the Linnean Society. 2010;99(1):1–8.

[12] Skelhorn J, Rowland HM, Speed MP, Ruxton GD. Masquerade: camouflage without crypsis. Science. 2010;327(5961):51-. doi: 10.1126/science.1181931 20044568 10.1126/science.1181931

[13] Barros B, Caetano JVO, Abrunhosa FA, Vallinoto M. Artisanal fisheries as indicator of troductivity in an Amazonian extractivist reserve (Curuçá River Estuary, NE Amazonian coast, Brazil). Journal of Coastal Research. 2011;64:1950–4.

[14] Barros B, Sakai Y, Hashimoto H, Gushima K, Vallinoto M. ‘Better off alone than in bad company’: agonistic colour display in mimetic juveniles of two ephippid species. Journal of Fish Biology. 2012;81(3):1032–42. doi: 10.1111/j.1095-8649.2012.03377.x 22880735 10.1111/j.1095-8649.2012.03377.x

[15] Queiroz AC, Sakai Y, Vallinoto M, Barros B. Morphometric comparisons of plant-mimetic juvenile fish associated with plant debris observed in the coastal subtropical waters around Kuchierabu-jima Island, southern Japan. PeerJ. 2016;4:e2268 doi: 10.7717/peerj.2268 27547571 10.7717/peerj.2268 PMC4974952

[16] Angermeier PL, Karr JR. Relationships between woody debris and fish habitat in a small warmwater stream. Transactions of the American Fisheries society. 1984;113(6):716–26.

[17] Randall JE. A review of mimicry in marine fishes. Zoological Studies. 2005;44(3):299.

[18] Carvalho LN, Zuanon J, Sazima I. The almost invisible league: crypsis and association between minute fishes and shrimps as a possible defence against visually hunting predators. Neotropical Ichthyology. 2006;4(2):219–24.

[19] Barros B, Sakai Y, Hashimoto H, Gushima K. Feeding behavior of leaf-like juveniles of the round batfish *Platax orbicularis* (Ephippidae) on reefs of Kuchierabu-jima Island, southern Japan. Journal of Ethology. 2008;26(2):287–93.

[20] Sazima I, Carvalho LN, Mendonça FP, Zuanon J. Fallen leaves on the water-bed: diurnal camouflage of three night active fish species in an Amazonian streamlet. Neotropical Ichthyology. 2006;4(1):119–22.

[21] Barros B, Sakai Y, Pereira PHC, Gasset E, Buchet V, Maamaatuaiahutapu M, et al Comparative allometric growth of the mimetic ephippid reef fishes *Chaetodipterus faber* and *Platax orbicularis*. PloS One. 2015;10(12):e0143838 doi: 10.1371/journal.pone.0143838 26630347 10.1371/journal.pone.0143838 PMC4668021

[22] Breder CM. On the behavior of young *Oligoplites saurus* (Bloch and Schneider). Copeia. 1942;1942(4):267-.

[23] Uchida K. Notes on a few cases of mimicry in fishes. Science Bulletin of the Faculty of Agriculture of Kyushu University. 1951;13:294–6.

[24] Randall JE, Randall HA. Examples of mimicry and protective resemblance in tropical marine fishes. Bulletin of Marine Science. 1960;10(4):444–80.

[25] Martin G, Pear JJ. Behavior modification: What it is and how to do it USA: Psychology Press; 2015.

[26] Masud R, Tsukamoto K. School formation and concurrent developmental changes in carangid fish with reference to dietary conditions When do fishes become juveniles?: Springer; 1998 p. 243–52.

[27] Ditty JG, Shaw RF, Cope JS. Distribution of carangid larvae (Teleostei: Carangidae) and concentrations of zooplankton in the northern Gulf of Mexico, with illustrations of early *Hemicaranx amblyrhynchus* and *Caranx spp*. larvae. Marine Biology. 2004;145(5):1001–14.

[28] Laroche WA, Ditty JG, Lamkin JT, Whitcraft SR. Carangida: Jacks In: Richards WJ, editor. Early stages of Atlantic fishes: an identification guide for the western central north Atlantic, Two Volume Set. 2: CRC Press; 2005 p. 1439–510.

[29] Able KW. A re-examination of fish estuarine dependence: evidence for connectivity between estuarine and ocean habitats. Estuarine, Coastal and Shelf Science. 2005;64(1):5–17.

[30] Sazima I, Uieda VS. Adaptações defensivas em jovens de *Oligoplites palometa* (Pisces, Carangidae). Revista Brasileira de Biologia. 1979;39(3):687–94.

[31] Sazima I. Deception, protection, and aggression in the mangrove: three juvenile fishes and floating leaves in Southeast Brazil. Aqua International Journal of Ichthyology. 2017;23(2):41–6.

[32] Bock WJ, Wahlert G. Adaptation and the form–function complex. Evolution. 1965;19(3):269–99.

[33] Douglas ME, Matthews WJ. Does morphology predict ecology? Hypothesis testing within a freshwater stream fish assemblage. Oikos. 1992:213–24.

[34] Scholtz G. Deconstructing morphology. Acta Zoologica. 2010;91(1):44–63.

[35] Norton SF, Luczkovich JJ, Motta PJ. The role of ecomorphological studies in the comparative biology of fishes Ecomorphology of fishes: Springer; 1995 p. 287–304.

[36] Loy A, Mariani L, Bertelletti M, Tunesi L. Visualizing allometry: geometric morphometrics in the study of shape changes in the early stages of the two-banded sea bream, *Diplodus vulgaris* (Perciformes, Sparidae). Journal of Morphology. 1998;237(2):137–46. 10.1002/(SICI)1097-4687(199808)237:2<137::AID-JMOR5>3.0.CO;2-Z 29852694

[37] Barros B, Sakai Y, Hashimoto H, Gushima K. Effects of prey density on nocturnal zooplankton predation throughout the ontogeny of juvenile *Platax orbicularis* (Teleostei: Ephippidae). Environmental Biology of Fishes. 2011;91(2):177–83.

[38] Giarrizzo T, Krumme U. Spatial differences and seasonal cyclicity in the intertidal fish fauna from four mangrove creeks in a salinity zone of the Curuçá estuary, north Brazil. Bulletin of Marine Science. 2007;80(3):739–54.

[39] Giarrizzo T, Krumme U. Temporal patterns in the occurrence of selected tropical fishes in mangrove creeks: implications for the fisheries management in north Brazil. Brazilian Archives of Biology and Technology. 2009;52(3):679–88.

[40] Gomes G, Sampaio I, Schneider H. Population Structure of *Lutjanus purpureus* (Lutjanidae-Perciformes) on the Brazilian coast: further existence evidence of a single species of red snapper in the western Atlantic. Anais da Academia Brasileira de Ciências. 2012;84(4):979–99. 23207703 10.1590/s0001-37652012000400013

[41] Silva R, Sampaio I, Schneider H, Gomes G. Lack of Spatial Subdivision for the Snapper *Lutjanus purpureus* (Lutjanidae–Perciformes) from Southwest Atlantic Based on Multi-Locus Analyses. PloS One. 2016;11(8):e0161617 doi: 10.1371/journal.pone.0161617 27556738 10.1371/journal.pone.0161617 PMC4996478

[42] Valentin AE, Penin X, Chanut JP, Sévigny JM, Rohlf FJ. Arching effect on fish body shape in geometric morphometric studies. Journal of Fish Biology. 2008;73(3):623–38.

[43] Abràmoff MD, Magalhães PJ, Ram SJ. Image processing with ImageJ. Biophotonics International. 2004;11(7):36–43.

[44] Klingenberg CP. MorphoJ: an integrated software package for geometric morphometrics. Molecular Ecology Resources. 2011;11(2):353–7. doi: 10.1111/j.1755-0998.2010.02924.x 21429143 10.1111/j.1755-0998.2010.02924.x

[45] Walker JA. Kinematics and performance of maneuvering control surfaces in teleost fishes. IEEE Journal of Oceanic Engineering. 2004;29(3):572–84.

[46] Gatz AJ. Community organization in fishes as indicated by morphological features. Ecology. 1979;60(4):711–8.

[47] Gibran FZ. Habitat partitioning, habits and convergence among coastal nektonic fish species from the São Sebastião Channel, southeastern Brazil. Neotropical Ichthyology. 2010;8(2):299–310.

[48] Adams DC, Otárola‐Castillo E. geomorph: an R package for the collection and analysis of geometric morphometric shape data. Methods in Ecology and Evolution. 2013;4(4):393–9.

[49] Anderson MJ, Gorley RN, Clarke KR. PERMANOVA+ for PRIMER: guide to software and statistical methods PRIMER-E: Plymouth, UK 2008.

[50] Team RC. R: a language and environment for statistical computing Vienna, Austria: R Foundation for Statistical Computing; 2017 Open access available at: http://cranr-projectorg. 2017.

[51] Clarke KR, Gorley RN. PRIMER v7: User Manual/Tutorial; PRIMER-E: Plymouth, UK 2015.

[52] Barros B, Higuchi H. Notes on morphological characters in early-developed *Monocirrhus polyacanthus* (Polycentridae, Perciformes). Kempffiana. 2007;3(2):18–22.

[53] Catarino MF, Zuanon J. Feeding ecology of the leaf fish *Monocirrhus polyacanthus* (Perciformes: Polycentridae) in a terra firme stream in the Brazilian Amazon. Neotropical Ichthyology. 2010;8(1):183–6.

[54] Kelley JL, Merilaita S. Testing the role of background matching and self-shadow concealment in explaining countershading coloration in wild-caught rainbowfish. Biological journal of the Linnean Society. 2015;114(4):915–28.

[55] Smith-Vaniz WF. Carangidae-Jacks and scads (also trevallies, queenfishes, runners, amberjacks, pilotfishes, pompanos, etc.) In: Carpenter KE, Niem VH, editors. FAO species identification guide for fishery purposes The living marine resources of the Western Central Pacific. 4. Bony fishes part 2 (Mugilidae to Carangidae). Rome: FOOD AND AGRICULTURE ORGANIZATION OF THE UNITED NATIONS; 1999.

[56] Hora SL. Ecology, bionomics and evolution of the torrential fauna, with special reference to the organs of attachment. Philosophical Transactions of the Royal Society of London Series B. 1930;218:171–282.

[57] Watson DJ, Balon EK. Ecomorphological analysis of fish taxocenes in rainforest streams of northern Borneo. Journal of Fish Biology. 1984;25(3):371–84.

[58] Leis JM. Is dispersal of larval reef fishes passive? In: Mora C, editor. Ecology of Fishes on Coral Reefs: Cambridge University Press; 2015 p. 223–6.

[59] Norcross BL, Shaw RF. Oceanic and estuarine transport of fish eggs and larvae: a review. Transactions of the American Fisheries Society. 1984;113(2):153–65.

[60] Boehlert GW, Mundy BC, editors. Roles of behavioral and physical factors in larval and juvenile fish recruitment to estuarine nursery areas American Fisheries Society Symposium; 1988.

[61] Jenkins GP, Black KP, Keough MJ. The role of passive transport and the influence of vertical migration on the pre-settlement distribution of a temperate, demersal fish: numerical model predictions compared with field sampling. Marine Ecology Progress Series. 1999:259–71.

[62] Castro JJ, Santiago JA, Santana-Ortega AT. A general theory on fish aggregation to floating objects: an alternative to the meeting point hypothesis. Reviews in Fish Biology and Fisheries. 2002;11(3):255–77.

